# The Effectiveness of a Bioactive Food Compound in the Lipid Control of Individuals with HIV/AIDS

**DOI:** 10.3390/nu8100598

**Published:** 2016-10-08

**Authors:** Rosângela dos Santos Ferreira, Rita de Cássia Avellaneda Guimarães, Elenir Rose Jardim Cury Pontes, Valter Aragão do Nascimento, Priscila Aiko Hiane

**Affiliations:** 1 Nutrition Service, University Hospital, Federal University of Mato Grosso do Sul—UFMS, Campo Grande 79079-900, MS, Brazil; 2 Post Graduate Program in Health and Development in the Central-West Region of Brazil, Federal University of Mato Grosso do Sul—UFMS, Campo Grande 79079-900, MS, Brazil; rita.guimaraes@ufms.br (R.d.C.A.G.); elenirpontes@uol.com.br (E.R.J.C.P.); aragao60@hotmail.com (V.A.d.N.); priscila.hiane@ufms.br (P.A.H.)

**Keywords:** lipid profile, anti-retroviral therapy, functional food, dyslipidemia, metabolic syndrome

## Abstract

Cardiovascular events due to decompensated lipid metabolism are commonly found in Human Immunodeficiency Virus/Acquired Immunodeficiency Syndrome (HIV/AIDS) patients using anti-retroviral therapy (HAART). Thus, the aim of this study was to identify the effect of a bioactive food compound (BFC) containing functional foods on individuals with HIV undergoing HAART. Particularly, this study aims to verify the clinical outcome in the change of the lipid profile due to the use of this compound. This study includes 115 individuals with HIV on HAART. All patients received dietary guidelines; however, sixty-one consumed BFC while fifty-one did not (NO BFC). Biochemical examinations and socio-demographic and clinical profiles were evaluated. As result, in patients using hypolipidemic and/or hypoglycemic drugs, there was 28.6% decrease in triglyceride levels (*p* < 0.001) in the NO BFC group, and 18.3% reduction in low density lipoprotein cholesterol (LDL-C) (*p* < 0.001) in the BFC group. In patients who did not use hypolipidemic and/or hypoglycemic drugs in the NO BFC group, there was 30.6% increase in triglycerides, 11.3% total cholesterol and 15.3% LDL-C levels (*p* < 0.001) while for the BFC group there was 4.5% reduction in total cholesterol (*p* < 0.001). In conclusion, this study evidenced that the dietary intervention containing BFC positively affected in lipid control, since these HIV/AIDS patients using HAART are more vulnerable to lipid disorders.

## 1. Introduction

Cardiovascular events resulting from a decompensated metabolism are commonly found among Human Immunodeficiency Virus/Acquired Immunodeficiency Syndrome (HIV/AIDS) patients undergoing Highly Active Antiretroviral Therapy (HAART). It results in an atherogenic lipid profile, which has the well-defined characteristics of chronic diseases [1,2]. In addition to dyslipidemia, there is also glucose intolerance, diabetes mellitus type II and elevated low density lipoprotein cholesterol (LDL-C), which are proven risk factors for cardiovascular diseases [3,4].

Most patients with coronary heart disease (CHD) have multiple lipid abnormalities. A management approach focusing exclusively on the decrease in LDL-C levels fails to address the related atherogenic potential that has been shown to exist when other CHD risk factors are present [5]. The condition known as mixed dyslipidemia (high LDL-C and triglyceride levels combined with decreased levels of high density lipoprotein cholesterol (HDL-C)) is commonly observed in patients with type II diabetes and metabolic syndrome (MetS) [6].

The increasing life expectancy, an aging population and high rates of smoking have led to concerns over the cardiovascular health of HIV-infected individuals in the long term [7]. Metabolic effects of HIV infection such as hypertriglyceridemia are long recognized. Side effects of HAART, such as dyslipidemia and insulin resistance, were described soon after its introduction [8].

In the absence of a specific consensus, the same criteria of the National Cholesterol Education Program (NCEP) for the general population without infections are recommended for the treatment of dyslipidemia in individuals undergoing HAART therapy [9,10]. Therefore, the adoption of healthy strategies following a nutritional control, including daily physical activity to increase muscle strength, adequacy of body mass and an improved lipid profile, especially LDL-C, triglycerides, very low density lipoprotein (VDVL-C) and total cholesterol [11,12], are recommended for HIV-infected individuals.

The ω-3 fatty acids are related to the decrease in hypertriglyceridemia by decreasing the activity of diacilglicerolaciltransferase (DGAT-1), an enzyme involved in the hepatic synthesis of triglycerides [13] decreasing the hepatic secretion of VLDL [14]. Moreover, because they are involved in important transcriptional regulatory pathways, they increase the peroxisome proliferator-activated receptor alpha (PPAR-α) involved in the synthesis of the lipoprotein lipase [15].

Polyunsaturated fatty acids (PUFA) modulate several genes involved in oxidative processes, increasing the expression of PPARs and blocking genes related to lipogenesis. Sterol regulatory element-binding proteins (SREBP) are transcription factors linked to the membranes that synthesize fatty acids. Their expression is decreased by polyunsaturated lipids [16].

The increase in HDL-C can be induced by restricting carbohydrates together with a decrease in body weight [17].

The lipid profile is also influenced by soluble fibers, form gels cross-linked with bile salts, forming micelles and changing the power of cholesterol resorption. Thus, the complex formed by bile-fiber is excreted through feces, decreasing the amount of bile acids in the enterohepatic cycle [18,19].

Another condition is the fiber’s ability to increase the growth of bifidobacteria and lactobacilli, which includes an improvement in the function of the intestinal barrier and the host’s immunity. It reduces subpopulations of potentially pathogenic bacteria, demonstrating a beneficial effect on the intestinal microbiota [20,21]. Another mechanism could be explained by an increase in the production of short chain fatty acids arising from the degradation of fibers, which inhibits the synthesis of hepatic cholesterol [22].

In a contemporary nutritional therapeutic approach, functional foods rich in fiber, especially soluble sources resulting from oat bran because it contains beta-glucan, and food sources from n-3 PUFA, such as flaxseed [23], are extremely important in the prevention of dyslipidemia since it exerts cardio-protective effects and minimizes the deposition of atheroma by mobilizing the accumulation of low density lipids in the walls of vessels and arteries, particularly those with foam characteristics [24].

Soy-based foods affect the lipid profile because their proteins and fibers decrease LDL-C and triglyceride levels, and increase HDL-C. The mechanism of action is related to the activity of soluble and insoluble fibers. Insoluble fibers are directly involved in the formation of stool [25].

The aim of this study was to identify the effect of Bioactive Food Compound (BFC) containing functional foods (oat bran, texturized soy protein and flaxseed) on individuals with HIV undergoing HAART. Particularly, this study aims to verify the clinical outcome of the change in the lipid profile due to the use of that compound.

It is hypothesized that this compound could be indicated with an adjuvant therapy to changes in the lipid profile of the population with HIV/AIDS presenting metabolic changes due to the continuous use of HAART.

## 2. Materials and Methods

### 2.1. Study Design and Participants

This is a prospective intervention study with 115 individuals with HIV on HAART using or not lipid-lowering and/or hypoglycemic medicaments. The subjects were selected from February 2011 to July 2012 at reference centers for treatment of HIV/AIDS in the state of Mato Grosso do Sul, Brazil. All study participants signed the consent term accepted by the Local Ethics Committee.

**Figure 1:**
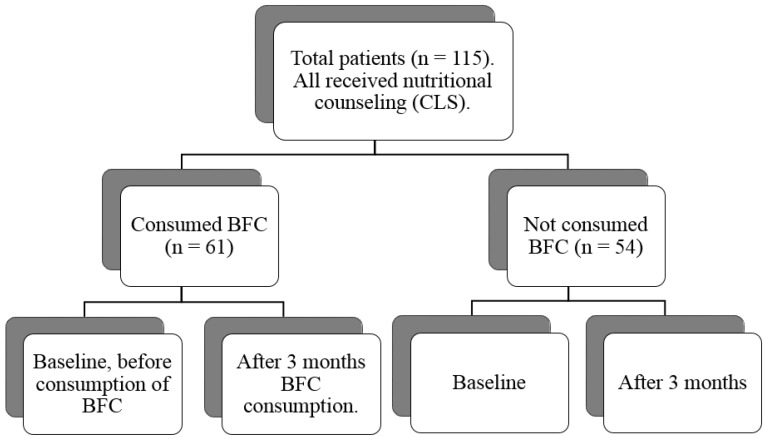
Study design. BFC= Bioactive Food Compound.

All patients received guidance on changes in lifestyle (CLS) during monthly ambulatory visits to the dietitian. The CLS consisted of (a) nutritional guidance on healthy eating, with control of total cholesterol and fractions, triglycerides and glucose; and (b) promotion of physical exercises (Figure 1).

Two groups were formed: individuals who consumed BFC (*n* = 61) and those who did not consume BFC (*n* = 54). Each member of the BFC group received three packages, with 1.2 kg each, containing the BFC for consumption during the study period and a consumption meter of the recommended daily dose (40 g). Patients were followed for three months, which enabled the comparison between baseline and after in each group (Figure 1).

### 2.2. Bioactive Food Compound (BFC)

The daily dosage of 40 g of BFC was prepared with the following composition: 20 g of oat bran, 10 g of textured soy protein and 10 g of crushed flaxseed in a 2:1:1 ratio. This formulation and quantity were defined by research on functional foods containing ingredients with different amounts and in isolation. The compound is registered at the National Industrial Property Institute (INPI) under No. BR 10 2013 018002 5 through the Intellectual Property Agency and Technology Transfer-APITT/CRE-Dean of Research, Graduate Studies and innovation/PROPP/Federal University of Mato Grosso do Sul, from July 2013, for patent application.

### 2.3. Biochemical Tests and Socio-Demographic and Clinical Profiles

Biochemical tests were performed at the beginning of the study and after 3 months. Particularly total cholesterol, HDL-C, LDL-C, triglycerides and fasting glucose were evaluated (references considered: total cholesterol <200 mg/dL; HDL-C for men, >55 mg/dL; for women, >65 mg/dL; LDL-C 130–159 mg/dL; triglycerides <150 mg/dL by the colorimetric method; fasting glucose (70–99 mg/dL) by the enzymatic method).

Data such as age, occupation (active and inactive), education level (elementary education, secondary education, higher education), regular physical activity (3 times a week for at least 30 min, categorized as yes and no), BMI (Body Mass Index), clinical classification in relation to dyslipidemia and metabolic syndrome (see Section 2.3.1), Classification of Antiretroviral Regimen (see Section 2.3.2), time of exposure to HAART and the use or non-use of lipid-lowering and/or hypoglycemic medicaments were collected.

#### 2.3.1. Classification of Dyslipidemia and Metabolic Syndrome

The dyslipidemia and metabolic syndrome were classified into the following groups (Table 1):

#### 2.3.2. Classification of Antiretroviral Regimen

The antiretroviral regimens were classified into the following groups:

- **Group I:** 2 NTRI + 1 PI or 2 NTRI + 1 NNTRI + PI;
- **Group II:** 2 NTRI + 2 PI (with ritonavir) or 2 NTRI + 1 NNTRI + 2 PI;
- **Group III:** 2 NTRI + 1 NNTRI.

### 2.4. Statistical Analysis

A comparison between the NO BFC and the BFC group was performed in relation to the study variables, the proportions by Chi-square test or Fisher’s exact test and the means by *t* test for independent samples. Then, the mean and standard deviation (SD) of BMI and biochemical variables were calculated before the beginning of the treatment with BFC and 3 months thereafter in the NO BFC and the BFC group, according to the use or the non-use of lipid-lowering and/or hypoglycemic medicaments. To compare the measurements in each group (before and after), the following tests were used: *t* test or Wilcoxon paired samples after checking the normal distribution by Lilliefor Test. The significance level of 5% was adopted.

### 2.5. Ethical Issues

The protocol conforms to the ethical guidelines of the 1075 Declaration of Helsinki. The Project was approved by the Local Ethical Committee (Federal University of Mato Grosso do Sul) under protocol No. 1630.

## 3. Results

### 3.1. Socio-Demographic, Biochemical and Clinical Profiles of the Population Analyzed

There was no difference in mean age between groups (*p* = 0.576 t test for independent samples), 48.8 years ± 8.5 (SD) in the BFC group and 47.8 years ± 10.4 (SD) in NO BFC, nor BMI at baseline (*p* = 0.132 *t* test for independent samples): the mean age was 25.5 ± 4.9 (SD) in the BFC group and 26.9 ± 5.1 (SD) in the NO BFC group.

According to Table 2, there was no significant difference between the BFC and the NO BFC groups in relation to gender, education level, occupation, physical activity, type and time of use of HAART therapy.

In clinical classification of dyslipidemia, which considers low HDL-C (<40 mg/dL for men and <50 mg/dL for women), defined as Group 4, the BFC group had a higher number of individuals with this type of decompensation (23%) compared to the NO BFC group (11.1%) (*p* = 0.023).

For the Group 2 of clinical classification of metabolic syndrome, which considers changes in normal values of total cholesterol (≤200 mg/dL), LDL-C (≤160 mg/dL), HDL-C (≥40 mg/dL for men and ≥50 mg/dL for women), triglycerides (≤150 mg/dL) and fasting glucose (≤100 mg/dL), there was a significant difference (*p* = 0.007). There was a higher percentage of individuals in the BFC with such disorder (26.2%) compared to the NO BFC group.

### 3.2. Biochemical Tests

In Table 3, when comparing the biochemical parameters of each group at the baseline and after 3 months, there was a significant increase in triglycerides (*p* < 0.001) for the NO BFC group, which was not using hypolipidemic and/or hypoglycemic drugs.

For the variable total cholesterol, in the same group (NO BFC), there was an increase in this fraction (*p* < 0.001). However, for the BFC group, a significant decrease in the serum lipid was reported (*p* = 0.021). In the NO BFC group, it was found a substantial increase in the LDL-C fraction (*p* < 0.001).

For the BFC group, which used hypolipidemic and/or hypoglycemic drugs, when comparing values before and after the use of bioactive compound, there was reduction of LDL-C levels (*p* = 0.043), and reduced triglycerides in the NO BFC group (*p* = 0.012).

### 3.3. Metabolic Response of the Bioactive Food Compound

Regarding the BMI in the BFC group, there was no statistical difference between the baseline and the value measured after three months, the mean was 25.5 ± 4.9 (SD) and 25.3 ± 4.7 (SD), respectively, (*p* = 0.203 *t* test for paired samples). In the NO BFC group, there was increase in BMI (*p* < 0.001 *t*-test for paired samples); the initial mean was 26.9 ± 5.1 (SD) and the final was 28.0 ± 5.1 (SD).

Table 4 shows individuals of the NO BFC group using medication for dyslipidemia. There was a decrease of 28.6% in triglyceride values. However, no significant differences were found in other parameters.

In the control group who did not use medication, there was significant increase of 30.6%, 11.3% and 15.3% in triglycerides, total cholesterol and LDL-C, respectively.

However, for the BFC group, which used medication, an 18.3% decrease in LDL-C was found 3 months after beginning the study. For those who used only BFC and did not use medication, there was a significant decrease of 4.5% in total cholesterol.

Based on the results, patients not taking hypolipidemic medication and not consuming the bioactive compound (BFC) showed increased levels of triglycerides, total cholesterol and LDL-C, whereas those who consumed BFC (also without medication) showed reduced total cholesterol.

## 4. Discussion

### 4.1. Socio-Demographic Profile and Clinical Classification of Dyslipidemia and Metabolic Syndrome of the INDIVIDUALS

The study population was considered homogeneous in terms of mean age, gender distribution, level of education, occupation and physical activity in groups (BFC and NO BFC), which is a positive aspect regarding the comparison between the groups.

Regarding classification of dyslipidemia, the BFC group had a higher percentage of HDL-C, but without clinical expression. This can be explained when a more systematic intervention by increasing the frequency and intensity of physical activity and a more specific dietary are not undertaken. There are cases of people with low HDL-C due to genetic problems, and they are more difficult to increase the level of this parameter [27]. There was higher percentage of patients of group 2 in the BFC group in the classification related to metabolic syndrome; however, the methodological procedure to measure biochemical parameters before and after each group studied has minimized possible limitations in the results analysis.

### 4.2. HAART and Metabolic Effects

In this study, there was no difference in relation to HAART time and type, since most of patients made use of antiretroviral regimen with protease inhibitors in the groups (BFC and NO BFC) for two years or more.

Domingos et al. [28] also stated that there was a predominance of patients on HAART therapy containing PI. Caramelli et al. [29] pointed out that, after the introduction of HAART containing PI, a remarkable increase in triglycerides and total cholesterol and a slight decrease in HDL-C were observed. The pathogenesis of dyslipidemia induced by the antiretroviral regimen is multifactorial and complex, involving several mechanisms triggered by the drugs of the treatment, besides the association with hormonal and immunological factors superposed in patients with a genetic predisposition [27].

The PI mechanism of action explains the homology between the catalytic region of the protease of HIV-1, the *C*-terminal region of the ligand cytoplasmic protein of retinoic acid type 1, named cytoplasmic retinoic acid binding protein type 1 (CRABP1), and the lipoprotein receptor-related protein (LRP). This change in the metabolism of the retinoic acid is due to the inhibition of cytochrome P450 isoenzymes by PIs, leading to a decrease in the differentiation and an increase in the apoptosis of peripheral adipocytes [30].

The interaction of PI’s catalytic site with LRP leads to a decrease in the cleavage of triglycerides into fatty acids and glycerol, a decrease in the hepatic uptake of chylomicron induced by central obesity, deposition of fat in the breasts and an increase in the peripheral resistance to insulin [31].

Evidence report that dyslipidemia associated with HAART accelerates the development of atherosclerosis and has a higher incidence of cardiovascular events, which is possibly related to the duration of the treatment [32,33,34].

### 4.3. Metabolic Response of BFC

The National Cholesterol Education Program Third Adult Treatment Panel [10] recommends that patients with borderline (150–200 mg/dL) levels of triglycerides be initially treated with changes in lifestyle. The NCEP ATP III also indicates that patients with high TG levels (200–499 mg/dL) should use pharmacologic therapy with statins, fibrates and nicotinic acid.

Triglycerides formed from long chain fatty acids from blood plasma or from lipid synthesis from acetyl coenzyme A (CoA). When increased, it may result in genetic changes or metabolic-potentiated by chylomicrons or VLDL-C atherogenic [35].

The high standard deviation (SD) obtained from some measurements of biochemical parameters was expected, since HIV/AIDS patients undergoing HAART are more vulnerable to lipid disorders. However, the methodological procedure to measure biochemical parameters before and after treatment in each group studied and the use of non-parametric statistical tests were used to minimize possible limitations in the results analysis.

In this study, BFC was effective in lipid control in patients who were not using hypolipidemic medication, but consumed the BFC for 3 months and reduced cholesterol levels. However, those who did not consume BFC had increased cholesterol, triglycerides and LDL-C.

The analysis becomes more relevant when all the parameters are analyzed together rather than separately. When hypolipidemic drugs are used, it is expected that the lipid levels will decrease or be maintained (plateau effect). In the absence of hypolipidemic medication and without the consumption of BFC, there was a lipid imbalance. This indicates the effectiveness of the compound. In other words, the performance of the compound in the daily diet is improved.

A study conducted by Ferreira et al. [36], who reported the nutritional quality indexes found in the BFC, showed the efficacy of fatty acids with respect to the reduction of atherogenic and thrombogenic events, since the compound contains flaxseed in its formulation. The significant reduction in LDL-C levels and total cholesterol levels is due to the functionality of fatty acids and soluble fiber, which act primarily in reducing atherogenic cholesterol, decreasing the deposition of atheroma in vein walls and arteries, thus preventing cardiovascular events, and consequently, factors associated with metabolic syndrome [37,38].

It is noteworthy that soy products are related to the prevention of atherosclerosis due to their antioxidant action on circulating lipids in plasma, as well as due to the presence of soluble fibers that act especially on the decrease of total cholesterol and LDL-C, since they reduce triglyceride levels besides exerting an anti-platelet aggregation effect [39,40,41].

The results of this study showed that BFC did not result in a significant decrease in glucose levels in the treatment group that did or did not use control medications, although soluble fiber present in the food compound has recognized anti-diabetic properties, especially by reducing intestinal absorption of cholesterol and carbohydrates and is therefore widely used in the control of diabetes [42]. However, Kim et al. [43] reported that the diet of diabetic mice foods high in fiber and polyunsaturated fatty acids resulted in decreased levels of triglycerides, total cholesterol and LDL-C, although it was not effective in improving glycemic control. However, Raimondi et al. [44] showed that nutritional counseling has a beneficial effect on the lipid profile and anthropometric parameters and the daily consumption of 40 g of oat bran provided an additional benefit in reducing insulin resistance parameters.

The study also showed that body weight was maintained after three months of BFC consumption, while there was weight gain in the group NO BFC. It is believed that the BFC may have produced a satiety effect as it contains dietary fiber, and this has the effect of increasing the volume without adding calories to diet and thus promotes weight loss [45].

## 5. Conclusions

A dietary intervention containing BFC positively affected lipid control, although the HDL-C and glucose levels were not affected, similar to the body weight because body mass index was not increased; that was positive to this data.

## Figures and Tables

**Table 1:** Classification of dyslipidemia and metabolic syndrome.

Group *	Categories	Criteria
A	1	isolated hypercholesterolemia (LDL-C ≥ 160 mg/dL)
	2	isolated hypertriglyceridemia (TG ≥ 150 mg/dL)
	3	mixed hypertriglyceridemia (increased LDL-C and TG)
	4	low HDL-C (<40 mg/dL for men, and <50 mg/dL for women)
B	1	low HDL-C (<40 mg/dL for men, and <50 mg/dL for women) and high total cholesterol (≥200 mg/dL)
	2	elevated total cholesterol (≥200 mg/dL), LDL-C (≥160 mg/dL), HDL-C (≤40 mg/dL for men, and ≤50 mg/dL for women), triglycerides (≥150 mg/dL) and fasting glucose (≥100 mg/dL)
	3	elevated fasting glucose levels (≥100 mg/dL)

* According to the V Brazilian Guideline on Dyslipidemia and Atherosclerosis Prevention (V BGDAP) [26]. LDL-C = low density lipoprotein cholesterol; HDL-C = high density lipoprotein cholesterol; TG = triglycerides.

**Table 2:** Socio-demographic and clinical profiles of patients and study variables.

Variables	BFC Group (*n* = 61)		NO BFC Group (*n* = 54)		*p*
	No.	%	No.	%	
Gender					
Female	34	55.7	25	46.3	0.312 ^(1)^
Male	27	44.3	29	53.7	
Education level					
Elementary	35	57.4	37	68.5	0.121 ^(1)^
Secondary	17	27.9	15	27.8	
Higher education	9	14.7	2	3.7	
Occupation					
Active	34	55.7	37	68.5	0.159 ^(1)^
Inactive	27	44.3	17	31.5	
Physical activity					
Yes	22	36.1	11	20.4	0.063 ^(1)^
No	39	63.9	43	79.6	
Clinical classification					
Dyslipidemia					
Group 1	3	4.9	3	5.6	1.000 ^(2)^
Group 2	22	36.1	29	53.7	0.464 ^(1)^
Group 3	3	4.9	9	16.7	0.107 ^(1)^
Group 4	14	23.0	6	11.1	**0.023** ^(1)^
Metabolic syndrome					
Group 1	1	1.6	-	-	0.470 ^(2)^
Group 2	16	26.2	6	11.1	**0.007** ^(1)^
Group 3	2	3.3	1	1.8	0.600 ^(2)^
HAART regimen					
Group I	6	9.8	2	3.7	0.307 ^(1)^
Group II	34	55.8	28	51.9	
Group III	21	34.4	24	44.4	
HAART time					
<2 years	10	16.4	14	25.9	0.209 ^(1)^
≥2 years	51	83.6	40	74.1	

^(1)^ Chi-square test; ^(2)^ Fisher’s exact test. BFC = Bioactive Food Compound.

**Table 3:** Biochemical variables of the groups and use of lipid-lowering and/or hypoglycemic medication.

Variables		BFC Group (*n* = 61)		NO BFC Group (*n* = 54)	
		Mean ± SD			
		Before	After	Before	After
**Without medication**	Triglycerides mg/dL	167.1 ± 78.7	174.4 ± 127.3	195.0 ± 66.9	254.7 ± 139.3
	*p* ^(2)^	0.484		**<0.001**	
	Cholesterol mg/dL	198.8 ± 35.8	189.8 ± 32.4	198.2 ± 55.2	220.6 ± 59.2
	*p* ^(1)^	**0.021**		**<0.001**	
	LDL-C mg/dL	119.9 ± 29.2	116.4 ± 29.4	124.6 ± 46.4	143.7 ± 53.4
	*p* ^(1)^	0.374		**<0.001**	
	HDL-C mg/dL	40.7 ± 14.1	43.6 ± 13.4	39.9 ± 17.4	42.8 ± 21.0
	*p* ^(1)^	0.101		0.068	
	Glucose mg/dL	87.3 ± 20.9	103.6 ± 101.7	89.5 ± 12.8	98.2 ± 44.4
	*p* ^(2)^	0.271		0.417	
**With medication**	Triglycerides mg/dL	232.9 ± 162.9	212.5 ± 145.6	279.0 ± 142.6	199.3 ± 107.1
	*p* ^(2)^	0.198		**0.012**	
	Cholesterol mg/dL	210.8 ± 80.3	188.9 ± 36.7	218.3 ± 45.7	183.4 ± 87.3
	*p* ^(1)^	0.199		0.266	
	LDL-C mg/dL	127.2 ± 62.5	103.9 ± 37.3	125.6 ± 33.9	136.6 ± 41.3
	*p* ^(1)^	**0.043**		0.248	
	HDL-C mg/dL	47.9 ± 15.2	46.4 ± 15.1	39.9 ± 9.0	47.7 ± 15.9
	*p* ^(1)^	0.569		0.075	
	Glucose mg/dL	107.4 ± 44.6	90.0 ± 11.8	138.2 ± 90.6	112.2 ± 37.7
	*p* ^(2)^	0.735		0.249	

^(1)^ *t* Test for paired samples; ^(2)^ Wilcoxon test. The *p* values in bold indicate a statistically significant difference (*p* ≤ 0.05). SD = standard deviation; BFC = Bioactive Food Compound; LDL-C = low density lipoprotein cholesterol; HDL-C = high density lipoprotein cholesterol.

**Table 4:** Comparison of biochemical variables in the BFC and NO BFC groups and use of lipid-lowering and/or hypoglycemic medication.

Variables (mg/dL)	BFC Group		NO BFC Group	
	Without Medication	With Medication	Without Medication	With Medication
Triglycerides	=	=	↑ 30.6%	↓ 28.6%
Total cholesterol	↓ 4.5%	=	↑ 11.3%	=
LDL-C	=	↓ 18.3%	↑ 15.3%	=
HDL-C	=	=	=	=
Glucose	=	=	=	=

↓ significant decrease between the initial measurement and the value measured after 3 months (*p* < 0.01); ↑ significant increase between the initial measurement and the value measured after 3 months (*p* < 0.01); = no significant difference between the initial measurement and the value measured after 3 months. BFC = Bioactive Food Compound; LDL-C = low density lipoprotein cholesterol; HDL-C = high density lipoprotein cholesterol.
